# Deterministic bead-in-droplet ejection utilizing an integrated plug-in bead dispenser for single bead–based applications

**DOI:** 10.1038/srep46260

**Published:** 2017-04-10

**Authors:** Hojin Kim, In Ho Choi, Sanghyun Lee, Dong-Joon Won, Yong Suk Oh, Donghoon Kwon, Hyung Jin Sung, Sangmin Jeon, Joonwon Kim

**Affiliations:** 1 Department of Mechanical Engineering, Pohang University of Science and Technology (POSTECH), 77 Cheongam-Ro, Nam-Gu, Pohang, Gyeongbuk 37673, Korea; 2 Department of Mechanical Engineering, Korea Advanced Institute of Science and Technology (KAIST), 291 Daehak-Ro, Yuseong-Gu, Daejeon 34141, Korea; 3 Department of Chemical Engineering, Pohang University of Science and Technology (POSTECH), 77 Cheongam-Ro, Nam-Gu, Pohang, Gyeongbuk 37673, Korea

## Abstract

This paper presents a deterministic bead-in-droplet ejection (BIDE) technique that regulates the precise distribution of microbeads in an ejected droplet. The deterministic BIDE was realized through the effective integration of a microfluidic single-particle handling technique with a liquid dispensing system. The integrated bead dispenser facilitates the transfer of the desired number of beads into a dispensing volume and the on-demand ejection of bead-encapsulated droplets. Single bead–encapsulated droplets were ejected every 3 s without any failure. Multiple-bead dispensing with deterministic control of the number of beads was demonstrated to emphasize the originality and quality of the proposed dispensing technique. The dispenser was mounted using a plug-socket type connection, and the dispensing process was completely automated using a programmed sequence without any microscopic observation. To demonstrate a potential application of the technique, bead-based streptavidin–biotin binding assay in an evaporating droplet was conducted using ultralow numbers of beads. The results evidenced the number of beads in the droplet crucially influences the reliability of the assay. Therefore, the proposed deterministic bead-in-droplet technology can be utilized to deliver desired beads onto a reaction site, particularly to reliably and efficiently enrich and detect target biomolecules.

Over the recent decades, bead-based assay methodologies have had a noticeable impact on the detection of biological and chemical analytes^1^,^2^. The surface of a microbead can be functionalized using various receptors (e.g., antibodies, peptides, DNA, mRNA, enzymes) and their compounds. By utilizing their mobility, functionalized microbeads can be used to effectively enrich and separate target analytes on the basis of affinity binding^3^,^4^,^5^. Furthermore, advances in bead-synthesis technologies^6^ and the development of precise handling methods for beads and samples have enabled single-molecule target detection (e.g., single-molecule PCR on microbeads^7^). To improve reproducibility and sensitivity of detection, many studies have focused on transferring a controlled number of particles (e.g., microbeads and biological cells) into a reaction chamber or onto a desired site^8^. To enhance the detection limit and to determine sample heterogeneity (e.g., detection of mutations), Prakash and Kaler^9^ proposed an ultralow, bead-based pathogen-detection method that employs liquid dielectrophoresis. However, despite the development of such technologies as inkjet and microfluidic chip-based particle dispensing, repeatable and precise control of the number of beads transferred into the reaction containers remains a challenge.

Inkjet-based particle dispensing has been widely used as it enables on-demand and noncontact delivery to various substrates and containers without the risk of cross-contamination. In this technique, the printing suspension is prepared through dilution to control the number of particles in a dispensed droplet^10^,^11^,^12^. Although this approach can produce a droplet encapsulating low numbers of particles, its performance in terms of regulating the number of particles is fundamentally limited by the Poisson distribution. Recently, many strategies that integrate inkjet dispensing with real-time particle detection have been demonstrated. In most of these strategies, the electrical signals (i.e., capacitance and impedance) from particles flowing near the dispensing nozzle is measured; a particle-encapsulated droplet is then ejected when the detected particle enters the dispensing volume^13^,^14^,^15^,^16^,^17^. Alternative methods include the automated optical counting of particles inside a region of interest near a dispensing nozzle and the sorting of droplets depending on the number of particles encapsulated in each droplet through mechanical movement of the stages^18^,^19^. However, these methods are not sufficiently efficient owing to inaccurate synchronization between the detection and dispensing modules.

In this paper, we propose a plug-in bead dispenser that uses a deterministic bead-in-droplet ejection (BIDE) mechanism through which a bead is actively transferred into the dispensing liquid volume via a microfluidic pneumatic valve^20^,^21^. The bead-encapsulated droplet is then ejected after the bead is transferred. By effectively integrating the dispensing and microfluidic particle-handling technologies, the number of beads in an ejected droplet is programmatically regulated for a given dispensing droplet volume. To the best of our knowledge, this is the first study to develop and demonstrate a single-bead dispensing technique in a deterministic manner. Furthermore, for reversible integration between a dispenser and a liquid cartridge, our previously developed plug–socket method that uses a macro-to-micro connector was adopted. This strategy offers disposability and easy replacement of the dispenser^22^. In this study, our proposed system was characterized under various operating conditions to realize automatic and deterministic BIDE. Finally, we demonstrated an application of the proposed bead-dispensing system by conducting a reliable bead-based bioassay for capturing and enriching target molecules.

## Materials and Methods

### System and operation

**Figure 1:**
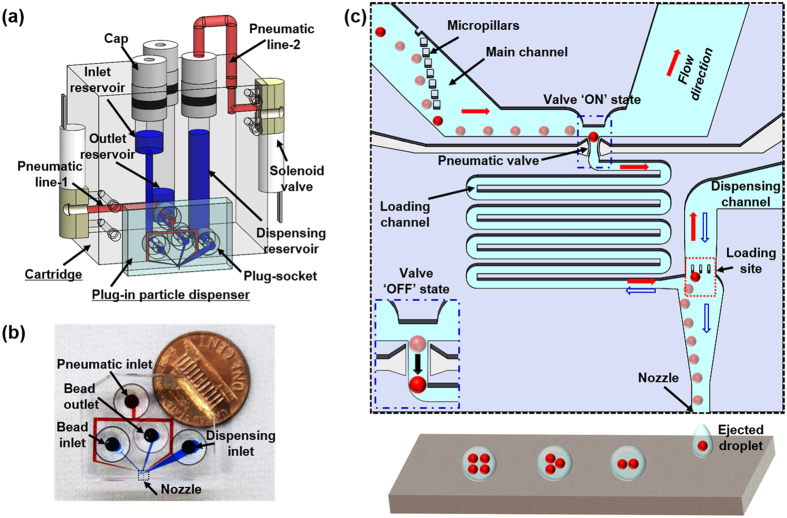
Design of the plug-in bead dispensing module. (**a**) Schematic of the dispensing module; (**b**) top view of the plug-in bead dispenser; (**c**) schematic of the deterministic BIDE mechanism and an enlarged view of the plug-in bead dispenser indicated in (**b**) by the black-dashed lines. Red and blue arrows indicate flow direction in normal and dispensing modes, respectively.

The plug-in bead-dispensing module is composed of a liquid cartridge and a plug-in bead dispenser. The cartridge comprises female sockets, liquid reservoirs, detachable caps, and solenoid valves (Fig. 1a), and the plug-in bead dispenser comprises a pneumatic inlet, a bead inlet and outlet, a dispensing inlet, a dispensing nozzle, and four male plugs assembled onto the back of each inlet and outlet (Fig. 1b). On the basis of our experience in a previous study, the plug–socket connection technique was employed to allow easy and reversible sealing against fluid flow and air compression^22^. The inlet reservoir, dispensing reservoir, outlet container, and pneumatic line 1 are connected to the bead inlet of the plug-in dispenser, the dispensing inlet, bead outlet, and pneumatic inlet via the plug–socket connection, respectively. Compressed air is supplied to the inlet, outlet, and dispensing reservoirs to induce fluid flow into the microchannel inside the dispenser. Compressed air is also supplied to pneumatic line 1 to operate the pneumatic valve of the dispenser.

The deterministic BIDE mechanism (Fig. 1c) consists of four operation modes: trapping, releasing, loading, and dispensing. The desired number of beads can be transferred on demand from the main channel onto the loading site through the pneumatically switchable valve, which serves the dual function of trapping and releasing a single bead. The red (solid) and blue (empty) arrows indicate the flow direction during the processes for loading a bead (i.e., trapping, releasing, and loading modes) and dispensing a bead-encapsulating droplet (i.e., dispensing mode), respectively. In the trapping mode, beads migrate laterally against the flow direction along the micropillars to follow the trapping stream, which enters the loading channel, leading to an effective inflow into the bead trap from the main channel^23^. On the basis of the dynamic change in hydraulic resistance, the valve containing a bead prevents the trapping of additional beads^20^. In the releasing mode, the trapped bead is released from the trap by switching the valve OFF, and the valve is rapidly closed to block the inflow of additional beads from the main channel. In the loading mode, the released bead is transferred onto the loading site via the loading channel. Thus, the number of beads within the dispensing volume area (i.e., loading site) can be regulated by controlling the number of times the valve is switched. In the dispensing mode, the loaded beads are ejected with a droplet by applying a positive pressure in the dispensing reservoir. This sequential operation of the four modes results in the ejection of deterministic bead-encapsulated droplets.

### Design and fabrication of dispensing module

The plug-in bead-dispensing module comprises a liquid cartridge and a plug-in dispenser. The acrylic cartridge is machined via computer numerical control (CNC) milling. The four female sockets at the front of the cartridge are 5.0 mm deep, with outer and inner diameters of 7.0 and 3.0 mm, respectively. A through-hole path (diameter 1 mm) was fabricated at the center of each socket to form the liquid channel connecting the liquid reservoirs and the dispenser. To prevent the sedimentation of the introduced beads because of gravity, the female socket connected to the inlet reservoir has a through-hole path that is smaller (diameter 0.5 mm) than that of the other sockets. Two three-way air solenoid valves (LHD series, Lee product) were mounted on the left and right sides of the liquid cartridge. One of the valves applies a pressure pulse to actuate the microfluidic pneumatic valve in the dispenser, and the other applies a pressure pulse to dispense the liquid.

A replica molding technique^24^ was utilized to fabricate the plug-in bead dispenser from polydimethylsiloxane (PDMS). It has two layers: a male plug to connect to the female socket on the liquid cartridge, and a microchannel that connects to the fluidic paths (Fig. 1b). The male plug was fabricated from an acrylic mold (depth 5.0 mm and inner and outer diameters 6.5 and 2.8 mm, respectively) through CNC milling. A mixture of the PDMS prepolymer and curing agent (10:1, w/w) was poured on the mold and was cured in an oven at 60 °C for 2 h. The plug-patterned PDMS layer was removed from the mold. At the center of each plug, a hole was punched using a biopsy punch (diameter 1.0 mm) to form the fluidic channels. The master mold (height 40 μm) for the microchannel was fabricated through photolithography by using a negative photoresist (KMPR 1025, MicroChem Corp.). The design and dimensions of the dispenser are illustrated in Supplementary Figs S1 and S2. The fabrication process was the same as that for the male plug, except that the PDMS mixture ratio was 14:1 (w/w) and that the mold was cured in the oven at 100 °C for 15 min. The two layers were then bonded through O2 plasma treatment at 45 W and 500 mTorr for 35 s. The assembly was left for 24 h at room temperature to ensure a permanent bond between the layers and to recover the mold’s surface property (i.e., hydrophobicity). Finally, a self-built tool22 was used to define the nozzle of the dispenser by using a microscope for precise alignment.

### Bead suspensions

A polystyrene bead suspension (diameter 25 μm, CV < 2%, Sigma-Aldrich) was diluted with a phosphate-buffered saline solution (PBS, Gibco) containing 0.5% (v/v) Tween 20 (Sigma-Aldrich) surfactant to reduce bead aggregation and nonspecific binding between the PDMS surface and polystyrene beads. For the dispensing test, suspensions of 50,000, 200,000, and 600,000 beads/mL were prepared. To effectively visualize the dispensed beads in the ejected droplet, a suspension of fluorescent beads (diameter 25 μm, CV < 0.8%, Microparticles) in deionized (DI) water with 1% (v/v) Tween 20 was used. For the streptavidin–biotin binding test, streptavidin-coated polystyrene beads (mean diameter 24.7 μm, Spherotech) were mixed with the DI water, and suspensions of 100,000 and 600,000 beads/mL were used, and a 100-μM Cy3-labelled biotin solution (Bioneer) was diluted with DI water to prepare 4, 8, 16, 32, 64, and 128 nM biotin solutions.

### Experimental setup

The experimental setup consists of a plug-in bead dispensing system, which includes a dispensing module, a pneumatic pressure controller, a three-axis manual stage, and a data acquisition device (NI-DAQ USB-6251, National Instruments), and an *in-situ* monitoring system, which includes a high-speed camera (Mini AX 100, Photron) with a X20 objective lens (M Plan Apo 20, Mitutoyo) and a 150 W halogen light source (Supplementary Fig. S3). A pneumatic controller is used to apply five pressure (P) levels (one negative and four positive: −100 kPa ≤ P ≤ 100 kPa, resolution 0.1 kPa) by using the minidiaphragm pumps (NMP 830 and 850 series, KNF Neuberger Ltd.) placed under the pressure sensors. The solenoid valves and LabVIEW software are used to electromechanically switch the pressure applied on the microfluidic pneumatic channel and the dispensing reservoir. All fluorescent images were captured using an inverted fluorescent microscope (IX-73, Olympus).

### Preloading of the dispensing module

Before all experiments, liquid reservoirs of the dispensing module and microfluidic liquid channels were filled with the intact PBS solution through micropumping and without any bubble trapping that could cause malfunctioning^25^. First, the dispenser and dispensing module were assembled, following which the outside of the nozzle was sealed using tape. Subsequently, approximately 5 μL of the PBS solution was let into each reservoir, and the assembly was placed in a vacuum chamber for approximately 15 min. The preloaded solution in the reservoirs percolates into the microchannel through the micropump because of the gas solubility of PDMS. Finally, the surfactant-treated PBS solution (0.5% (v/v) Tween 20) was loaded into the liquid reservoirs.

### Streptavidin–biotin binding test

To investigate the capturing performance of biotin molecules as a function of the number of beads in a given reaction volume, we first dispensed droplets encapsulating different numbers of streptavidin beads on the cover glass via the proposed bead dispensing system to dispense single beads and the manual pipetting to dispense multiple beads, respectively. After evaporation of the droplet, the streptavidin beads remained; then, the Cy3-labelled biotin solution was manually pipetted onto the streptavidin beads. The streptavidin bead surface was enriched with biotin molecules through internal flow and diffusion during evaporation. Finally, the buffer droplet was put on the reacted bead to wash excessive residue not bound to the streptavidin bead, following which the emitted fluorescence signal was measured. This procedure was repeated with 4, 8, 16, 32, 64, and 128 nM biotin solutions and with different numbers of streptavidin beads. Two hundred nanoliters of streptavidin bead suspension with different concentrations of 100,000 and 600,000 beads/mL was manually pipetted to load different numbers of beads on each cover glass; on average, 12.4 (SD: ±3.6, n = 6) and 79.4 (SD: ±33, n = 6) beads were loaded at these two concentrations, respectively.

## Results and Discussion

### Trapping, releasing, loading, and dispensing of a single bead

**Figure 2:**
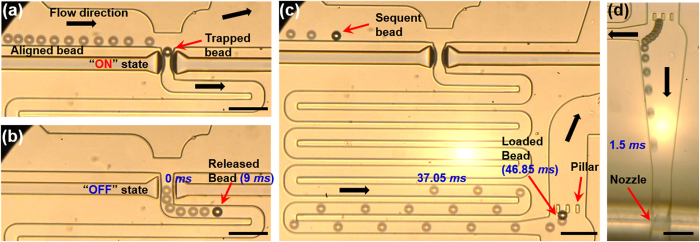
Time-lapse images for each mode. (**a**) Trapping mode during the ON state of the pneumatic valve; (**b**) releasing mode during the OFF state of the pneumatic valve; (**c**) loading mode to transfer a bead onto the loading site defined by the micropillars; (**d**) dispensing mode to eject a bead-encapsulated droplet. (**a**–**c**) were captured at 4,000 fps, and (**d**) was captured at 10,000 fps. All scale bars are 100 μm.

The trapping, releasing, loading, and dispensing modes were observed through *in-situ* monitoring using a high-speed camera (Fig. 2). In the trapping mode, the beads introduced from the inlet reservoir were aligned using micropillars to allow them to flow into the trapping stream^26^ (Fig. 2a). These pillars can effectively align the beads without additional force (e.g., sheath flow and magnetic phoresis^27^), thereby simplifying dispenser design and operation. A single bead was successfully trapped by utilizing dynamic changes in hydraulic resistance and trapping space, as described in our previous work^20^. Once the trap is filled with a bead, the hydraulic resistance of the pneumatic valve part increases, reducing the width of trapping stream. Sufficient reduction of the trapping stream can prevent subsequent beads from entering the trap. The trapping condition of a single bead was experimentally characterized as described in following the ‘optimization for trapping and releasing single beads’ section. In the releasing mode, the pneumatic valve was switched OFF for 15 ms to release the trapped bead (Fig. 2b), after which it was immediately turned ON again, because a longer OFF time could induce the additional inflow of beads from the main channel. The released bead flowing through a sinuous channel was loaded onto the loading site defined by the micropillars, which are 20 μm apart, a spacing smaller than the bead diameter (25 μm; Fig. 2c). The bead travelled from the pneumatic valve to the loading site in a few dozen milliseconds. After loading a bead onto the loading site, a bead-encapsulated droplet was formed at the nozzle by positively pressurizing the dispensing reservoir, which is negatively pressurized during the normal state (Fig. 2d). To transfer sufficient energy for forming a droplet against the capillary force, a positive pressure of 80 kPa was applied to the dispensing reservoir for 30 ms. The bead on the loading site rushed toward the nozzle because the sinuous channel was designed to have sufficient hydraulic resistance to backflow. Thus, a bead-encapsulated droplet was finally obtained through these sequential modes. We name this technology “deterministic bead–in-droplet ejection (BIDE).”

### Optimization for trapping and releasing single beads

**Figure 3:**
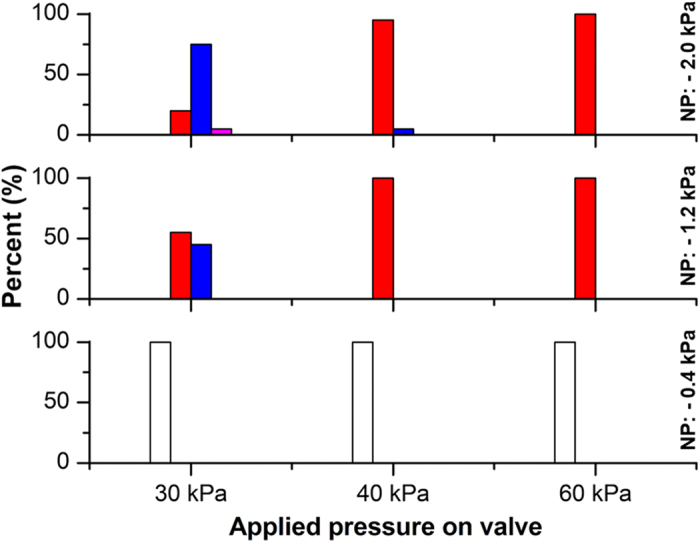
Optimization test result for trapping single bead. At various negative pressures in the dispensing reservoir and positive pressures on the pneumatic valve, the number of trapped beads was observed 20 times under each condition. NP indicates negative pressure. Black-empty, red-solid, blue-solid, and magenta-solid bars indicate 0, 1, 2, and 3 beads, respectively.

The operating conditions for trapping and releasing single beads were optimized to ensure that only a single bead reached the loading site. According to reported experimental results^20^, trapping of a single bead can be achieved by controlling the width of the trapping stream at the branch point and the trapping space. In the present experiment, the trapping stream was regulated by controlling the pressures applied to the inlet, outlet, and dispensing reservoirs. Pressures were set to 2 and 1.2 kPa at the inlet and outlet, respectively, to minimize the consumption of the bead suspension and to prevent beads from sinking to the bottom of the inlet reservoir and the plug channel. In the dispensing reservoir, a negative pressure was normally applied to retract the residual liquid into the nozzle in order to prevent the wetting of the nozzle after droplet ejection and to efficiently attract released beads from the trap to the loading site. To experimentally optimize the trapping stream, the amplitude of the negative pressure was varied from −0.4 to −2.0 kPa. The maximum amplitude was limited to −2 kPa because at higher amplitudes, the meniscus of the liquid at the distal end of the nozzle retracts, trapping bubbles in the dispenser. Such trapped bubbles could induce malfunctions in each operation mode by altering the hydraulic resistances in the channels where the bubbles are trapped. Next, the trapping space was regulated by deflecting the pneumatic valve; this deflection is dependent on the magnitude of the applied positive pressure. The number of trapped beads at various negative pressures in the dispensing reservoir and positive pressures on the pneumatic valve was analyzed (Fig. 3).

After preloading of the dispensing module, 30 μL of the bead suspension with 50,000 beads per mL was carefully introduced into the inlet reservoir. Pipetting a small amount of the suspension—rather than completely filling the inlet reservoir—results in economical consumption of the bead suspensions, which are expensive. At a negative pressure of −0.4 kPa, the beads did not flow into the trapping site, indicating that the trapping stream is thinner than the diameter of the bead aligned with the side of the wall (Fig. 2a). The bead flows along the streamline as a low-Reynolds-number laminar flow. To increase the volume of the trapping stream, the magnitude of the negative pressure in the dispensing reservoir was gradually increased to −1.2 kPa and −2 kPa, and the consequent trapping of the beads at the pneumatic valve was monitored. Moreover, similar to other trapping parameters, trapping space varied with pressure supplied to the pneumatic valve. The maximum applied positive pressure was restricted to 60 kPa to ensure appropriate operation of the valve and a firm plug–socket connection: the burst pressure of the connection at which the air leaks is approximately 80 kPa. Multiple bead trapping was occasionally observed at pressures less than 60 kPa. According to our results, the optimum pressure at the inlet, outlet, and dispensing reservoir was 2, 1.2, and −2 kPa, respectively, and 60 kPa at the pneumatic valve for trapping and releasing single beads.

### Single bead–in-droplet ejection frequency

For practical application of the proposed bead-dispensing system, the entire process was automated without microscopic observation to validate the observed trapping, releasing, loading, and dispensing processes. A one-click operation was performed using LabVIEW software to trigger the electro-pneumatic switching valves (i.e., the solenoid valve). We first defined the required times for each operation mode. Images from the high-speed camera shown in Fig. 2 demonstrate that the releasing, loading, and dispensing modes each lasted only tens of milliseconds. Thus, the required time for the entire process was analyzed on the basis of trapping time, which lasted several seconds.

**Figure 4:**
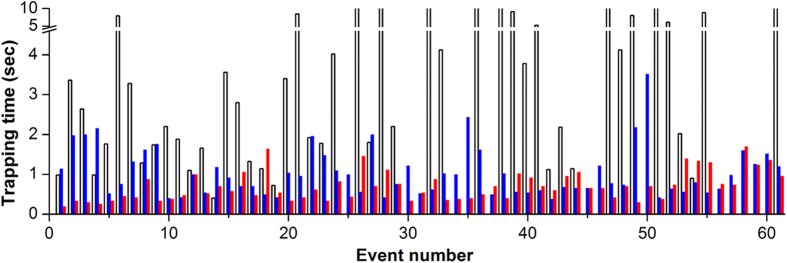
Trapping time variation at different bead concentrations. Red-solid, blue-solid, and black-empty bars indicate 600,000, 200,000, and 50,000 beads/mL, respectively.

Trapping time is defined as the time until the trapping of a following bead after the release of a trapped bead. Therefore, trapping time depends on the flux of the influent beads and is thus a function of the flow rate and bead concentration. We measured the trapping time at various bead concentrations at the optimized operation condition (i.e., at constant flow rate). To reduce operating time, we followed “trap–release–dispense–load” mode sequence rather than the “trap–release–load–dispense” sequence (Supplemental video clip 1). This change in the sequenced enabled faster reloading (by tens of milliseconds) of the following bead on the loading site; it also minimizes the time required for the entire process by enabling parallel operation of the modes: during continuous ejections, loading and trapping can occur concurrently. After a simple feasibility test, we ran the trapping test under the assumption that trapping a single bead requires less than 10 s. The time for each mode were set as follows: 10 s for trapping, 15 ms for releasing, and 30 ms for dispensing. The entire process was continually repeated for approximately 15 min (i.e., 90 droplet ejections). After 3 min, by which time the beads pipetted into the inlet reservoir reach the bead trap in the dispenser, the data for the trapping time was plotted (Fig. 4). As the bead concentration increased, the trapping time tended to decrease. At 50,000 beads/mL, the average trapping time was 6.5 s (standard deviation (SD) 10.22 s, maximum (MAX) 60 s, minimum (MIN) 4 s). Trapping failure occurred frequently (18%). The average trapping time was 1.05 s (SD 0.62 s, MAX 3.52 s, MIN 0.38 s) at 200,000 beads/mL and 0.71 s (SD 0.37 s, MAX 1.7 s, MIN 0.2 s) at 600,000 beads/mL. Although the trapping time decreased as bead concentration increased, a higher concentration occasionally resulted in channel clogging. Defect-free operation (i.e., 100% dispensing of droplets, each of which encapsulated a single bead) was realized when the trapping time was set sufficiently longer than the measured maximum trapping time for each concentration. At 600,000 beads/mL, we chose a trapping time of 3 s, considering the maximum trapping time of 1.7 s and an approximately 2-fold safety factor. Under the optimized operating conditions, our plug-in bead dispensing system was used for more than 300 ejections of droplets encapsulating a single bead, thereby confirming defect-free operation (Supplemental video clip 2).

### Dispensing a droplet with a controlled number of beads

**Figure 5:**
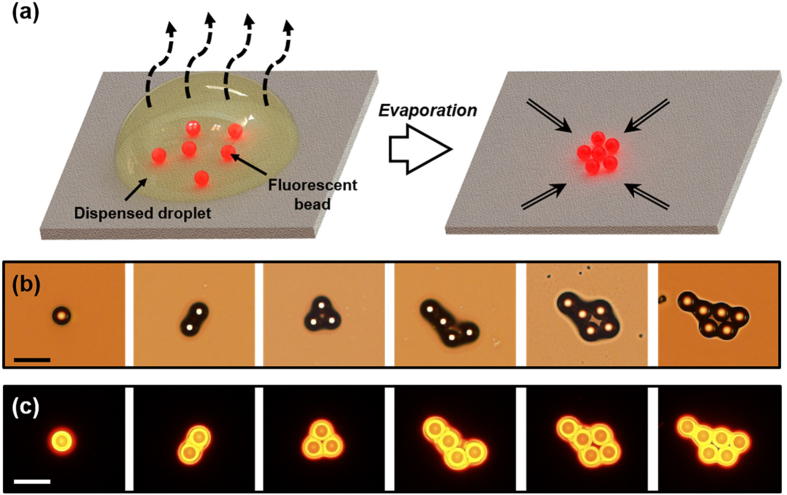
Visualization of beads in a dispensed droplet. (**a**) Schematic of beads aggregated during evaporation for effectively detecting and counting beads; (**b**) bright-field microscopy images; (c) fluorescent microscopy images of beads after evaporation. All scale bars are 50 μm.

To the best of our knowledge, this is the first report of a deterministic bead-in-droplet technology to accurately control the encapsulated bead number at low bead numbers (i.e., less than 10 beads) and within a fixed droplet volume. By repeatedly switching the pneumatic valve, the desired number of beads was loaded sequentially and ejected onto a cover glass. Beads in a dispensed droplet were aggregated during its evaporation, and the phenomenon facilitated the detection and counting of dispensed beads (Fig. 5a, and supplemental video clip 3). For effective bead counting and visualization, red fluorescent beads were used; a 1% (v/v) Tween 20–DI water solution was used as the buffer solution because ion crystals, which disturb bead aggregation, form during evaporation of the PBS solution. Figure 5b,c demonstrate bright-field images and fluorescent images of bead aggregates after evaporation of the dispensed droplets encapsulating a single bead to up to six beads, respectively. In case of more than 10 beads, a few beads moved toward the loading channel in the dispensing mode and not toward the nozzle. This problem can be solved through effective design of the loading site by increasing of the length (i.e., hydraulic resistance) of the loading channel and by a comprehensive analysis of streamline and bead trajectories in the dispensing mode.

### Biotin–streptavidin binding via droplet evaporation

**Figure 6:**
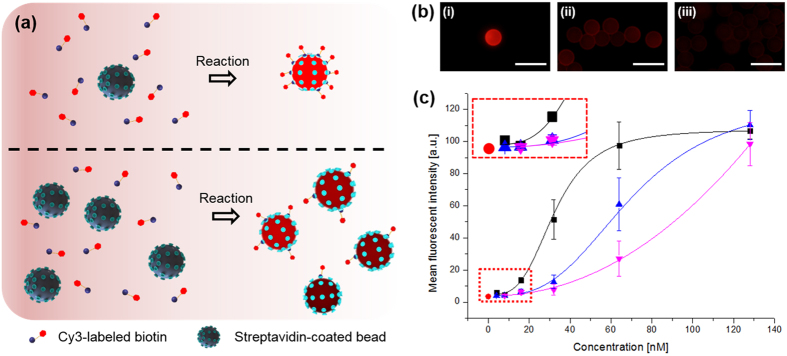
Bead-based streptavidin–biotin binding test. (**a**) Schematic of the collision and binding phenomena between streptavidin-coated beads and biotin molecules depending on the number of beads; (**b**) representative fluorescence images with (i) single, (ii) 12.4 ± 3.6, and (iii) 79.4 ± 33.0 beads in a dispensed droplet; (**c**) MFI of single beads at different biotin molecule concentrations after reactions with different numbers of streptavidin beads (square, upper triangle, and lower triangle indicate single, 12.4 ± 3.6, and 79.4 ± 33.0 beads, respectively). All scale bars are 50 μm.

As a practical application of our bead dispensing system, affinity-based capturing and enrichment of biotin molecules was demonstrated using streptavidin-coated beads in an evaporating droplet. During droplet evaporation, collision and binding between biotin molecules and streptavidin beads occur effectively through diffusion and internal flow^28^. Here, we discuss the effect of the number of streptavidin beads in an evaporating droplet on the detection limit and sensitivity. We assumed that collision of a streptavidin bead against biotin molecules would be more frequent with decrease in the total number of streptavidin beads loaded in a droplet; this led to strong enrichment of the biotin molecules (Fig. 6a). Fluorescence images of beads in pipetted PBS solution were captured after the interaction between the Cy3-labeled biotin solution and the streptavidin bead in an evaporating droplet (Fig. 6b). Complete evaporation of 200 nL of biotin solution required approximately 10 min under ambient conditions (temperature 25 °C and humidity 40%). Figure 6c shows the mean florescent intensity (MFI) per bead at different bead numbers. At 128 nM, the capturing performance (i.e., MFI) was reasonably similar. MFIs are in saturation for beads in a dispensed droplet because an adequate number of biotin molecules are spread around each bead. However, at concentrations less than 128 nM, a decline in the capturing performance per bead was observed, because the predispensed number of beads increases even under the same concentration, which validates the foregoing assumption. We anticipate that this feature would help enhance the detection limit and sensitivity in bead-based assays, such as the Luminex technology, based on single-bead detection^29^. In addition, highly reliable results are expected through precisely controlled bead numbers and repeatable experiments.

## Conclusion

A deterministic bead-in-droplet dispensing system with a plug-in microfluidic bead dispenser was developed through the effective integration of on-demand droplet dispensing and microfluidic particle trap-and-release technologies. For easy and reversible integration of the dispensing module, the plug-and-socket connecting method was used. Furthermore, the operating conditions (inlet, outlet, dispensing, and valve pressures) of the system were optimized, and minimal consumption of a 30 μL bead suspension was realized to facilitate economical operation. The entire process of the deterministic BIDE mechanism was automated using a programmed sequence, without any visual observation, for increasing the practicability of the proposed technique. Using a 600,000 beads/mL bead suspension, bead-encapsulated droplets were continuously ejected every 3 s for an optimized interval (i.e., 10 min) without any failure to dispense single beads. In addition, the encapsulated number of beads in a dispensing droplet was deterministically regulated by appropriate switching of the microfluidic valve. Finally, an ultralow, bead-based streptavidin–biotin binding test via droplet evaporation demonstrated the novel, sensitive, and reliable detection of target molecules by using the proposed technique. With advances in synthesis and functionalities of beads, the proposed bead dispensing system can be applied to improve various parameters (e.g., reliability, sensitivity, and limit of detection) of bead-based applications.

## Additional Information

**How to cite this article:** Kim, H. *et al*. Deterministic bead-in-droplet ejection utilizing an integrated plug-in bead dispenser for single bead–based applications. *Sci. Rep.* **7**, 46260; doi: 10.1038/srep46260 (2017).

**Publisher's note:** Springer Nature remains neutral with regard to jurisdictional claims in published maps and institutional affiliations.

## Supplementary Material

Supplementary Information

Supplementary video 1

Supplementary video 2
