# Supplementary material for: Deterministic bead-in-droplet ejection utilizing an integrated plug-in bead dispenser for single bead–based applications
Source: Sci Rep. 2017 Apr 10;7:46260. doi: 10.1038/srep46260 (PMC5385560; doi:10.1038/srep46260)

SUPPLEMENTARY MATERIAL

Deterministic bead-in-droplet ejection utilizing an integrated plug-in bead dispenser for single bead-based applications

Hojin Kim[a]†, In Ho Choi[a]†, Sanghyun Lee[a], Dong-Joon Won[a], Yong Suk Oh[b], Donghoon Kwon[c], Hyung Jin Sung[b], Sangmin Jeon[c] and Joonwon Kim[a*]

[a]Department of Mechanical Engineering, Pohang University of Science and Technology (POSTECH), 77 Cheongam-Ro, Nam-Gu, Pohang, Gyeongbuk 37673, Korea

[b]Department of Mechanical Engineering, Korea Advanced Institute of Science and Technology (KAIST), 291 Daehak-Ro, Yuseong-Gu, Daejeon 34141, Korea

[c]Department of Chemical Engineering, Pohang University of Science and Technology (POSTECH), 77 Cheongam-Ro, Nam-Gu, Pohang, Gyeongbuk 37673, Korea

†Hojin Kim and In Ho Choi contributed equally to this work.

[*]Corresponding author

Tel: +82-54-279-2185
Fax: +82-54-279-2960
E-mail: Joonwon@postech.ac.kr

1. The design and dimensions of the plug-in bead dispenser

(a) The top view of the dispenser

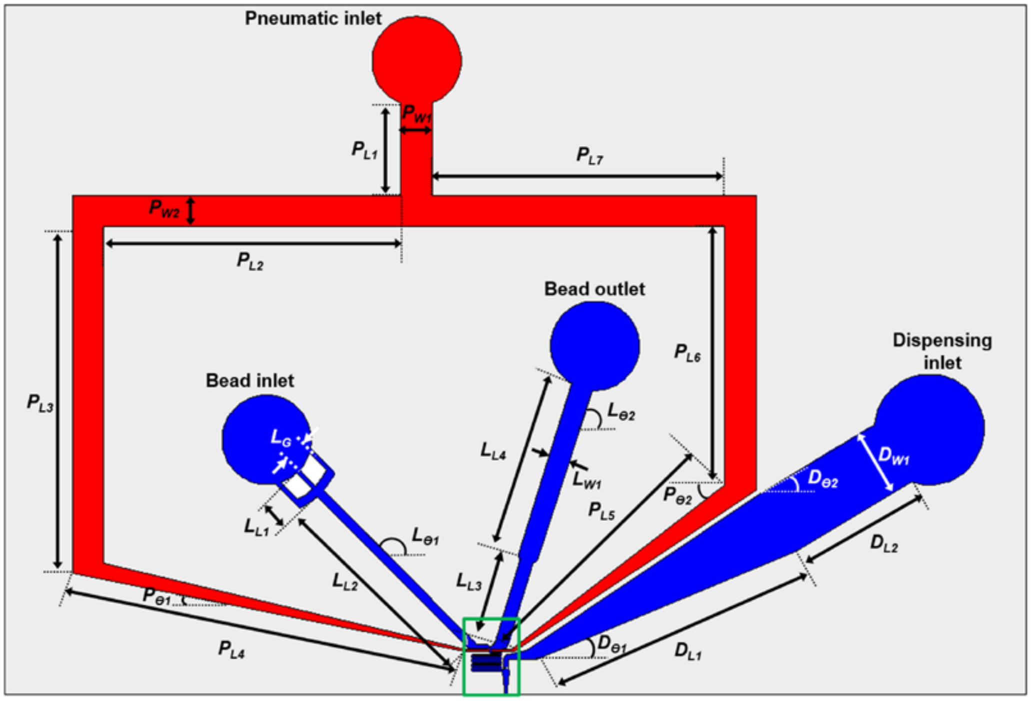

Figure S1. The top view of the dispenser

Table S1. Summary of dispenser dimensions in figure S1

Pneumatic channel	Dimensions	Bead channel	Dimensions	Dispensing channel	Dimensions
P_{L1}	2.067 mm	L_{L1}	0.698 mm	D_{L1}	6.306 mm
P_{L2}	6.658 mm	L_{L2}	4.802 mm	D_{L2}	3.000 mm
P_{L3}	7.530 mm	L_{L3}	2.000 mm	$D_{\theta 1}$	23 °
P_{L4}	8.951 mm	L_{L4}	4.062 mm	$D_{\theta 2}$	31 °
P_{L5}	6.029 mm	L_{G}	0.408 mm	D_{W1}	1.516 mm
P_{L6}	5.792 mm	$L_{\theta 1}$	135 °	-	-
P_{L7}	6.545 mm	$L_{\theta 2}$	72 °	-	-
P_{W1}	0.725mm	L_{W1}	0.443 mm	-	-
P_{W2}	0.700 mm	-	-	-	-
$P_{\theta 1}$	11 °	-	-	-	-
$P_{\theta 2}$	38 °	-	-	-	-

(b) An expanded view of the green box in figure S1

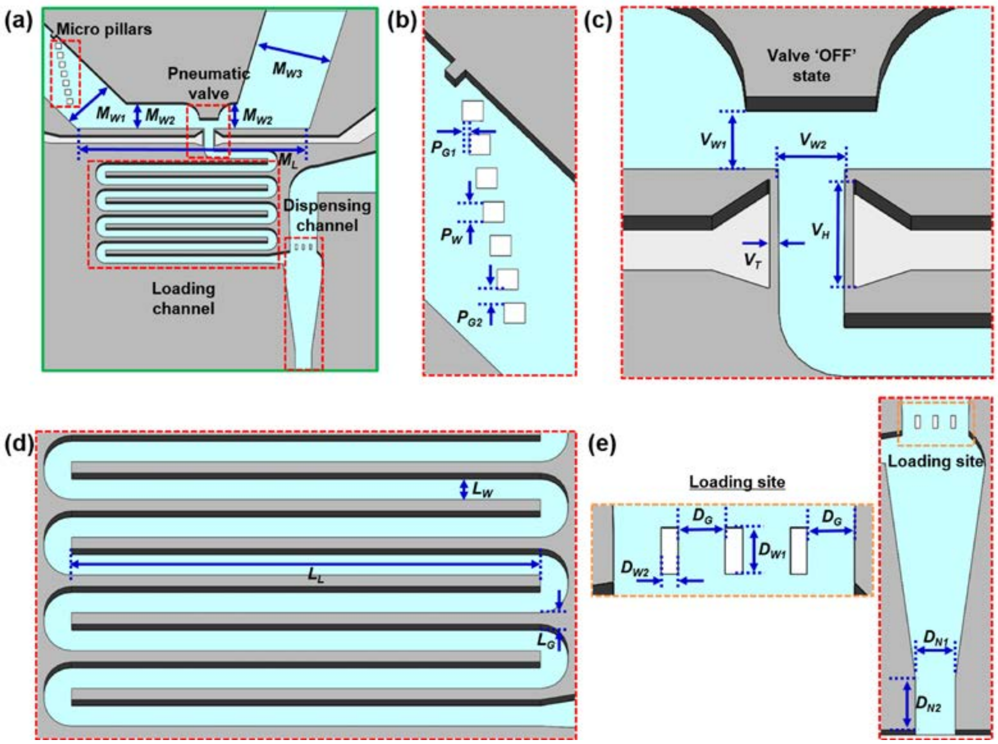

Figure S2. Details of the dispenser in an expended view. (a) overall view, (b) micro pillars, (c) pneumatic valve, (d) loading channel, and (e) dispensing channel with the loading site.

Table S2. Summary of the dispenser dimensions in figure S2

**Main channel (a)**	M_{W1}	200 µm
	M_{W2}	100 µm
	M_{W3}	300 µm
	M_{L}	860 µm
**Micro pillars (b)**	P_{G1}	7 µm
	P_{G2}	14 µm
	P_{W}	21 µm
**Pneumatic valve (c)**	V_{W1}	40 µm
	V_{W2}	35 µm
	V_{T}	5 µm
	V_{H}	60 µm
**Loading channel (d)**	L_{W}	35 µm
	L_{L}	600 µm
	L_{G}	15 µm
**Dispensing channel (e)**	D_{W1}	20 µm
	D_{W2}	7 µm
	D_{G}	20 µm
	D_{N1}	60 µm
	D_{N2}	100 µm

2. Details of experimental setup

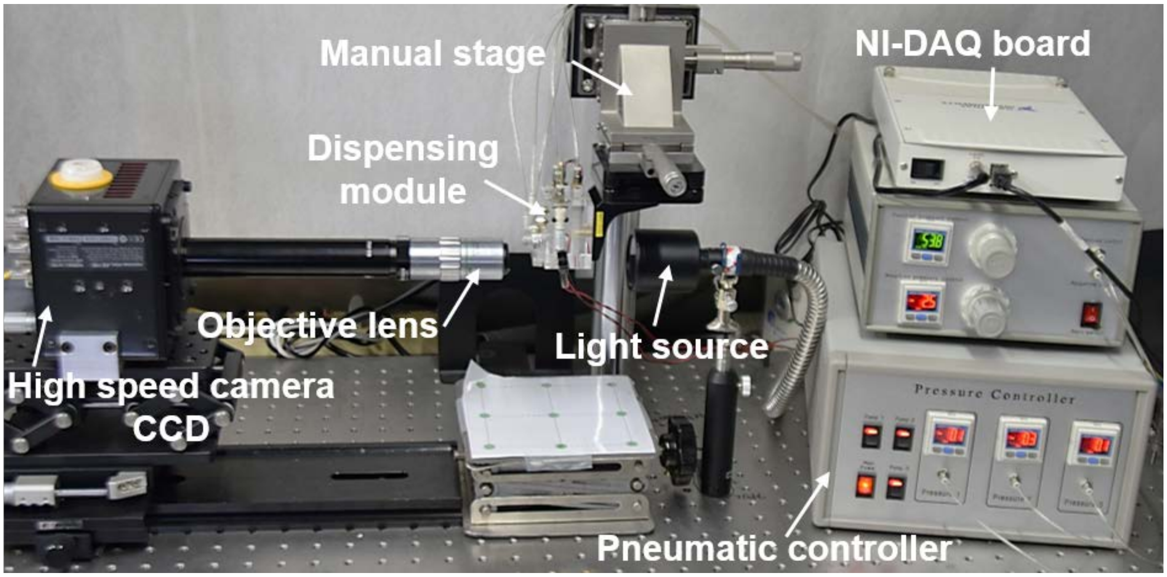

Figure S3. Experimental setup.

3. Video clip 1 showing the defect-free operation of the deterministic BIDE at the optimized operating conditions (captured by a high-speed camera at 500 fps)

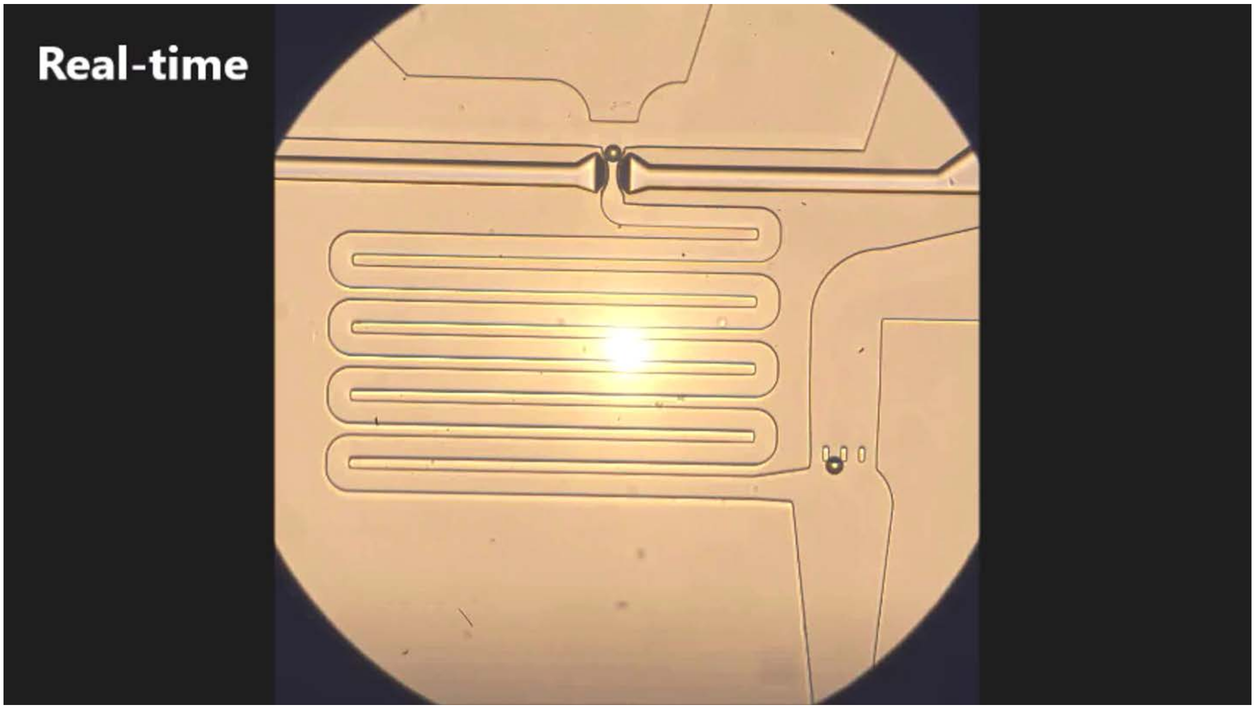

4. Video clip 2 showing the aggregation of four fluorescent beads in an evaporating droplet

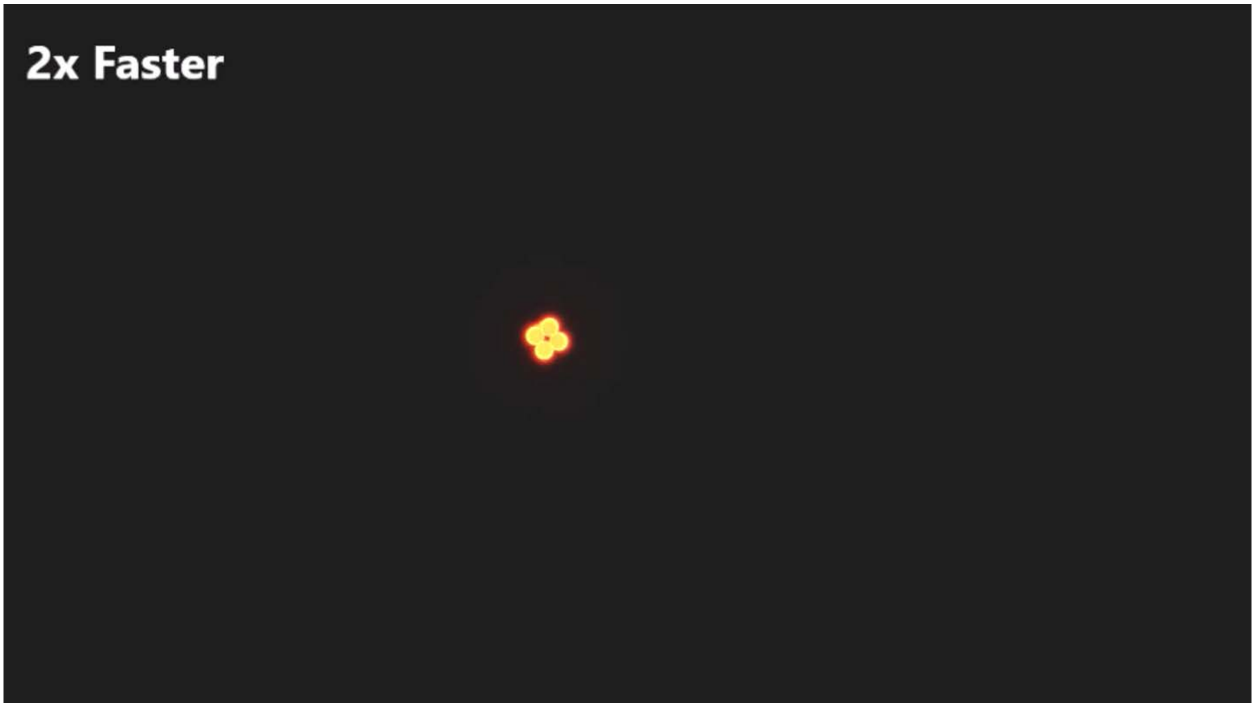

Supplement: Supplementary Information [file srep46260-s1.pdf]
